# Allelic Heterogeneity and Genetic Modifier Loci Contribute to Clinical Variation in Males with X-Linked Retinitis Pigmentosa Due to *RPGR* Mutations

**DOI:** 10.1371/journal.pone.0023021

**Published:** 2011-08-12

**Authors:** Abigail T. Fahim, Sara J. Bowne, Lori S. Sullivan, Kaylie D. Webb, Jessica T. Williams, Dianna K. Wheaton, David G. Birch, Stephen P. Daiger

**Affiliations:** 1 Human Genetics Center, School of Public Health, University of Texas Health Science Center at Houston, Houston, Texas, United States of America; 2 Retina Foundation of the Southwest, Dallas, Texas, United States of America; 3 Department of Ophthalmology, University of Texas Southwestern Medical Center, Dallas, Texas, United States of America; Innsbruck Medical University, Austria

## Abstract

Mutations in *RPGR* account for over 70% of X-linked retinitis pigmentosa (XlRP), characterized by retinal degeneration and eventual blindness. The clinical consequences of *RPGR* mutations are highly varied, even among individuals with the same mutation: males demonstrate a wide range of clinical severity, and female carriers may or may not be affected. This study describes the phenotypic diversity in a cohort of 98 affected males from 56 families with *RPGR* mutations, and demonstrates the contribution of genetic factors (i.e., allelic heterogeneity and genetic modifiers) to this diversity. Patients were categorized as grade 1 (mild), 2 (moderate) or 3 (severe) according to specific clinical criteria. Patient DNAs were genotyped for coding SNPs in 4 candidate modifier genes with products known to interact with RPGR protein: *RPGRIP1*, *RPGRIP1L*, *CEP290*, and *IQCB1*. Family-based association testing was performed using PLINK. A wide range of clinical severity was observed both between and within families. Patients with mutations in exons 1–14 were more severely affected than those with ORF15 mutations, and patients with predicted null alleles were more severely affected than those predicted to make RPGR protein. Two SNPs showed association with severe disease: the minor allele (N) of I393N in *IQCB1* (p = 0.044) and the common allele (R) of R744Q in *RPGRIP1L* (p = 0.049). These data demonstrate that allelic heterogeneity contributes to phenotypic diversity in XlRP and suggest that this may depend on the presence or absence of RPGR protein. In addition, common variants in 2 proteins known to interact with RPGR are associated with severe disease in this cohort.

## Introduction

Retinitis pigmentosa (RP) is a form of inherited retinal degeneration characterized by progressive loss of photoreceptors. Initially rods are affected and patients present with decreased night vision and mid-peripheral visual field defects. In advanced disease, cones are affected as well and patients progress to macular involvement and eventual blindness. The genetic etiology of RP is exceptionally diverse. Over 40 genes are currently known to cause RP, many displaying allelic heterogeneity, and some demonstrating both dominant and recessive modes of inheritance (RetNet; http://www.sph.uth.tmc.edu/retnet/). Clinical phenotypes are equally diverse in terms of age of onset, clinical severity, rate of progression, degree of cone involvement, and presence of additional sequelae such as macular edema. In particular, X-linked RP (RP3 [MIM 300029]) demonstrates marked variable expressivity among both affected males, with a wide range of severity, and carrier females, who may or may not have clinical symptoms [1], [2].

Theories to explain this phenotypic diversity include differences in the disease-causing mutation, genetic modifiers, and environmental modifiers. Over 70% of XlRP cases are due to mutations in retinitis pigmentosa GTPase regulator (*RPGR* [MIM 312610]), which localizes to the connecting cilium in photoreceptors and is thought to play a role in protein transport [3], [4], [5]. XlRP demonstrates considerable allelic heterogeneity: more than 300 different *RPGR* mutations have been found to date in families with XlRP (http://rpgr.hgu.mrc.ac.uk). Therefore, clinical variability may be due, in part, to different disease-causing mutations.

This prediction is consistent with the remarkable complexity of *RPGR* splicing, which gives rise to at least 10 different *RPGR* splice variants in a tissue-specific manner [6], [7], [8], [9]. Therefore the pathologic effect of a particular mutation may depend on its presence or absence in the most prominent retinal splice variants. For example, an estimated 60% of disease-causing mutations in *RPGR* are found in ORF15, an alternatively spliced exon including exon15 and extending into intron 15 [7]. The ORF15-containing *RPGR* transcript is abundant in human retina and has been demonstrated as the predominant isoform present in mouse photoreceptor connecting cilia [3], [7]. The number of different RPGR protein isoforms expressed in the retina is unclear. One study showed only two isoforms in human retina, one detectable with a C-terminal antibody specific for the canonical RPGR_1–19_ (containing exons 1–19), and the other detectable with a C-terminal exon against the ORF15 alternative exon [10]. Another study using similarly-placed antibodies showed three different bands for RPGR _1–19_ and five different bands for RPGR_ORF15_, suggestingting that the various bands could be due to post-translational modification (i.e., different isotypes) or to alternate splicing (i.e., different isoforms) [11].

Prior studies have found evidence that different types of *RPGR* mutations cause different phenotypes [12], [13], [14], [15], [16]. In particular, patients with mutations in exons 1–14 demonstrated smaller visual fields than patients with mutations in ORF15 [12]. In addition, several families with cone-rod dystrophy have been reported with mutations in *RPGR*, all of them in ORF15 downstream of ORF15 codon 445 [13], [14], [15], [16]. There is also evidence that some mutations in *RPGR* are more likely to be penetrant in female carriers while other mutations cause disease only in hemizygous males [1], [17], [18].

However, allelic heterogeneity alone cannot fully account for the observed phenotypic variability in XlRP caused by *RPGR* mutations. In one report describing two families with the same disease-causing mutation arising independently, female carriers in one family had no clinical symptoms while females in the other family were severely affected [19]. Even members of the same family can have striking phenotypic differences [20],[21]. These observations strongly suggest the presence of genetic and environmental modifiers.

This study aimed to categorize the phenotypic diversity in affected males from 56 families with mutations in *RPGR*, and to determine the contribution of genetic factors (i.e. allelic heterogeneity and genetic modifiers) to this diversity. Ninety-eight affected males with 44 different *RPGR* mutations were included. To examine genotype-phenotype correlations, mutations were categorized by the physical location in the gene as well as by the predicted biologic outcome. A candidate modifier-gene approach was taken to screen genes meeting the following criteria: 1) the protein is known to interact with RPGR, 2) the protein has polymorphic amino acid substitutions, and 3) the gene contains known retinal disease-causing mutations. Therefore, coding SNPs in RPGR-interacting protein 1 (*RPGRIP1* [MIM 605446]) [22], [23], [24], [25], RPGRIP1-like (*RPGRIP1L* [MIM 610937]) [26], [27], [28], centrosomal protein 290 kDa (*CEP290* [MIM 610142]) [29], [30], [31], [32], [33], [34], [35], [36], and IQ motif containing B1 (*IQCB1* [MIM 609237]; aka nephrocystin-5) [37] were chosen for analysis. This study demonstrates significant *RPGR* genotype-phenotype correlations and reports two SNPs in candidate modifier genes associated with disease severity.

## Methods

### Patients and clinical assessment

Patients with mutations in *RPGR* were ascertained from the Southwest Eye Registry (SER) at the Retina Foundation of the Southwest (RFSW) [38]. The SER is a database of over 3,000 patients referred to the RFSW for retinal degenerative diseases. Ninety-eight affected males from 56 families were ascertained. This study was performed in accordance with the Declaration of Helsinki and informed consent was obtained from all participants. The research was approved by the Committees for Protection of Human Subjects at the University of Texas, Health Science Center at Houston and the University of Texas Southwestern Medical Center.

For each patient, manifest refraction and best-corrected visual acuity were assessed with the NIKON RETINOMAX using the EVA-ETDRS visual acuity chart. Humphrey visual fields were obtained using a model 740 Humphrey field analyzer. Program 30-2 was used to determine static parametric thresholds at 74 locations within the central 30 degrees. Frequency domain optical coherence tomography (fdOCT) retinal scans were obtained using a Heidelberg Spectralis OCT. Fundus photos were obtained with a Canon digital camera (CF-60UD) and included a posterior pole view and additional peripheral views to document bone spicules and vessel attenuation if present. To assess dark-adaptation, pupils were maximally dilated using 1.0% cylcopentolate hydrochloride and 2.5% phenylephrine hydrochloride, followed by 45 minutes of dark adaptation. The final dark-adapted threshold was determined using an 11-degree test stimulus located 7 degrees below fixation on a Goldmann-Weekers dark-adaptometer.

Full-field electroretinograms (ffERG) were obtained according to ISCEV standards using Burian-Allen electrodes. Signals were elicited for rod response, mixed rod-cone response, and cone response, and signals were amplified and computer-averaged. Multi-focal electroretinograms (mfERG) were obtained using the Veris system, and responses were recorded from 103 locations within the central 40 degrees.

Fifty-four of the 98 affected males had 3 or more visits at least 1 year apart. For these individuals, log cone 31 hz flicker ERG amplitude was plotted as a function of patient age. Linear regression analysis was used to define a.) the log amplitude at birth (age 0) and b.) the slope of the best-fit regression line. The regression line could then be used to determine the predicted amplitude at age 18, even for those patients who were not tested at age 18. Predicted amplitude at age 18, therefore, depended on both the severity at birth (a) and the rate of progression (b). This analysis assumes a constant rate of progression in log amplitude versus age, that is, patients are predicted to lose a constant percentage of amplitude each year. The results of these regression analyses were consistent with this assumption, as are previous analyses of ERG longitudinal data [39], [40], [41].

The 54 patients with multiple visits were characterized as grade 1 (mild), grade 2 (moderate), or grade 3 (severe) primarily on the basis of the derived cone 31 hz flicker ERG amplitude at age 18, supplemented by Humphrey visual fields. Supplementary measures, along with cone ERG amplitude, were also used to characterize the 44 patients with a single visit. Criteria used to categorize patients as grade 1, 2 or 3 are summarized in Table 1.

**Table 1:** Criteria for assigning clinical categories to males with *RPGR* mutations.

Severity	ERG	Visual Field
Grade 1	≥10 µvolts 30 Hz flicker in teens (1–5 microvolts in 30's)	Good central 30° field sensitivity
Grade 2	1–5 µvolts 30 Hz flicker in teens	Marked central field constriction; some sensitivity beyond 20°
Grade 3	<1 µvolt 30 Hz flicker in teens/20's	Marked central field constriction

### RPGR mutation detection

RPGR mutations were identified or confirmed in-house, in every family, using our standard RPGR sequencing protocols [42]. Genomic DNA was amplified for 35 cycles with AmpliTaq Gold® 360 Master Mix (Applied Biosystems, Foster City, CA) and M13-tailed primers designed to flank exons 1–19. RPGR ORF15 was amplified for 40 cycles using AmpliTaq Gold® 360 Master Mix and the following primers: 5′GACTAAACCATAATATCCAAATCCA3′ and ′GCCAAAATTTACCAGTGCCTCCTAT3′. PCR product was treated with ExoSAPIT (Affymetrix, Santa Clara, CA) and sequenced using BigDye v1.1 (Applied Biosystems). Exons 1–19 were sequenced in two directions using M13 primers, and ORF15 was sequenced unidirectionally using a set of 7 nested primers. Sequence reactions were purified using BigDye Xterminator, run on a 3500 Genetic Analyzer., and analyzed using SeqScape v2.7 (Applied Biosystems). A minimum of one affected male was tested in each family.

### Haplotype analysis of *RPGRIP1* and *RPGRIP1L*

DNA samples from hybrid murine-human cell lines monosomic for either human chromosome 14 or 16 were a gift from Dr. James Hixson [43]. All coding SNPs in *RPGRIP1* and *RPGRIP1L*, with documented minor allele frequency of at least 1.0% in dbSNP or in the literature [22], [26], were sequenced in cell lines monosomic for human chromosome 14 or 16, respectively. Each SNP was sequenced in at least 100 cell lines, including 32–38 cell lines derived from each of three ethnicities: African-Americans (from Atlanta, GA), European-Americans (from Rochester, MN), and Mexican-Americans (from Starr County, TX). Exons in *RPGRIP1* and *RPGRIP1L* containing candidate SNPs were amplified from 80 ng of genomic DNA per reaction. PCR was performed using AmpliTaq Gold 360 (Applied Biosystems, Carlsbad, CA) in a 12.5 uL reaction, including 0.25 uL of GC enhancer (Applied Biosystems), primers at 0.2 uM each, and bovine serum albumin at 144 µg/mL. PCR conditions were as follows: 95°C for 15 minutes, 35 cycles of 95°C for 45 seconds, 56°C for 45 seconds, and 72°C for 45 seconds, followed by extension at 72°C for 5 minutes. Two uL of each PCR product was purified in a 4 uL reaction using ExoSapIt (USB, Cleveland, OH), and purified product was sequenced using BigDye v1.1 (Applied Biosystems) in both directions. For each sequencing reaction, 2 uL of purified PCR product was sequenced in a 5 uL reaction with 0.16 uM sequencing primer. Sequencing reactions were purified using BigDye Xterminator (Applied Biosystems) according to manufacturer's instructions and run on an ABI 3730 DNA Analyzer (Applied Biosystems). Sequence results were analyzed using SeqScape v2.6 (Applied Biosystems).

### Genotyping candidate modifier loci in patients

Blood samples were collected in EDTA-coated tubes and centrifuged at 3000×g for 10 minutes. The leukocyte-rich buffy coat interface was separated and stored at −80°C. DNA was later extracted using the Gentra Puregene blood kit (Qiagen, Valencia, CA). DNA sequences containing SNPs of interest were amplified from 37.5 ng genomic DNA per reaction with either AmpliTaq Gold 360 (Applied Biosystems) and the conditions described above, or PyroMark PCR Kit (Qiagen).

The PyroMark PCR reactions were performed for subsequent pyrosequencing, and therefore included two specific primers, one contained an M13 tail, and the other a universal biotinylated M13 primer, as described by Guo and Milewicz [44]. The final concentrations of the universal biotinylated M13 primer and the specific un-tailed primer were 0.2 uM each, and the final concentration of the specific primer with an M13 tail was 0.01 uM in a 12.5 uL reaction volume. PCR conditions for PyroMark reactions were as follows: 95°C for 15 minutes, 45 cycles of 94°C for 30 seconds, 52°C for 30 seconds, and 72°C for 30 seconds, followed by extension at 72°C for 10 minutes. Five uL of PCR product was added to 5 uL water, 2 uL streptavidin-sepharose beads (GE Healthcare Bio-Sciences AB, Uppsala, Sweden), and 38 uL PyroMark Binding Buffer (Qiagen), and vortexed for 30 minutes. PCR product was then captured on a PyroMark Vacuum Prep Tool (Qiagen) by aspiration according to manufacturer's instructions, and washed in 70% ethanol for 5 seconds, Pyromark Denaturation Solution (Qiagen) for 5 seconds, and PyroMark Wash Buffer (Qiagen) for 5 seconds. PCR product was then released into 15 uL of 0.3 uM sequencing primer diluted in PyroMark Annealing Buffer (Qiagen), heated at 70°C for 15 minutes, and cooled at room temperature for at least 10 minutes before running on a PSQ HS 96A Instrument (Qiagen).

### Data analysis

Genotype-phenotype correlations were analyzed using the Fisher Exact Test and generalized estimating equation (GEE) analysis. GEE analysis is a variance-minimization strategy that tests for significant associations in cohorts that are organized into clusters, such as families, and accounts for highly correlated phenotypes within a cluster that may be due to factors other than the variable being tested [45]. In this case, the “other factors” would be shared genes other than RPGR. GEE analysis accounts for this by performing a weighted analysis, in which the weight assigned to each individual is inversely proportional to the degree of correlation of phenotypes within that individual's family.

Family-based association testing was performed using PLINK (http://pngu.mgh.harvard.edu/purcell/plink/) [46]. Specifically, the DFAM procedure was used, which incorporates TDT using parent-child trios, sib-TDT using discordant sibships, and unrelated individuals, which are stratified into clusters and treated as sibships using the Cochran-Mantel-Haenszel test. In this analysis, which requires a dichotomous phenotype, patients with grade 1 (mild) or 3 (severe) RP (as defined above under “Patients and Clinical Assessment”) were compared, while patients with grade 2 (moderate) RP were excluded. Significance values were not corrected for multiple comparisons given the limited number of SNPs tested.

## Results

### A wide range of clinical consequences was observed both within and between families

**Figure 1:**
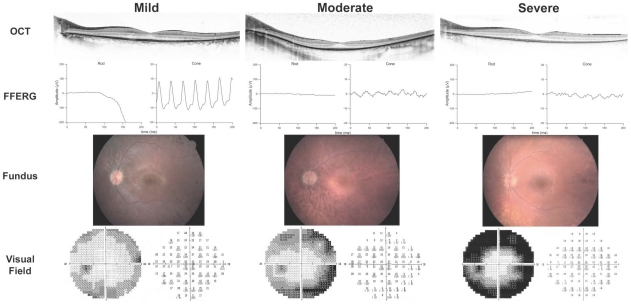
Representative images and studies for grade 1, 2, and 3 RP. Representative fdOCTs, ffERGs, fundus photos, and visual fields (OS) are shown for a male with an *RPGR* mutation in each of the three clinical categories. Mild = grade 1; Moderate = grade 2; Severe = grade 3.

**Figure 2:**
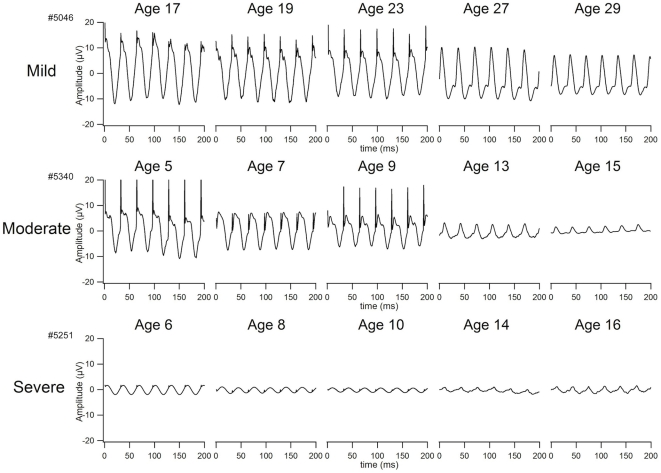
Representative ERGs for grade 1, 2, and 3 RP. Longitudinal cone 31 hz flicker ERGs are shown for a male with an *RPGR* mutation in each of the three clinical categories. Mild = grade 1; Moderate = grade 2; Severe = grade 3.

**Figure 3:**
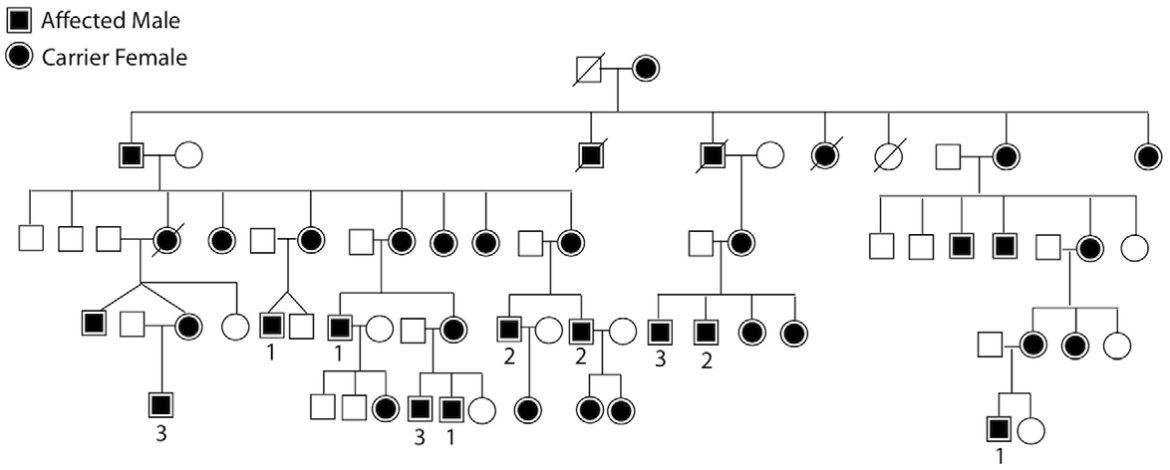
A large pedigree with a mutation in *RPGR* showing intrafamilial phenotypic variability. 1 = grade 1 (mild), 2 = grade 2 (moderate), 3 = grade 3 (severe).

Ninety-eight affected males from 56 families with mutations in *RPGR* were enrolled. Clinical work-up included best-corrected visual acuity, Humphrey visual fields, fdOCT, dark-adapted threshold, ffERG, mfERG, and fundus exam with photography. Patients were characterized as having grade 1 (mild), 2 (moderate), or 3 (severe) disease according to the guidelines in Table 1. Representative fundus photos, ERGs, and visual fields from each of these three categories are shown in Figure 1. For 54 of the 98 males, ERGs from multiple visits were available, and linear regression analysis was used to derive cone 31 hz flicker ERG amplitude at age 18. This information was used in assigning clinical severity according to the guidelines in Table 1. Figure 2 shows longitudinal cone ERG data from individuals with grades 1, 2, and 3 RP. The cohort included 21 grade 1, 34 grade 2, and 43 grade 3 individuals as defined by these standards. Of note, there were no unaffected males with known mutations, consistent with the fact that *RPGR* mutations have historically been fully penetrant in the hemizygous state. Although most pedigrees were small, with only one or two affected individuals ascertained, some of the larger pedigrees demonstrated intrafamilial phenotypic variability (Figure 3).

### Predicted null alleles are associated with more severe disease while alleles predicted to produce variant proteins are associated with more phenotypic variability

**Figure 4:**
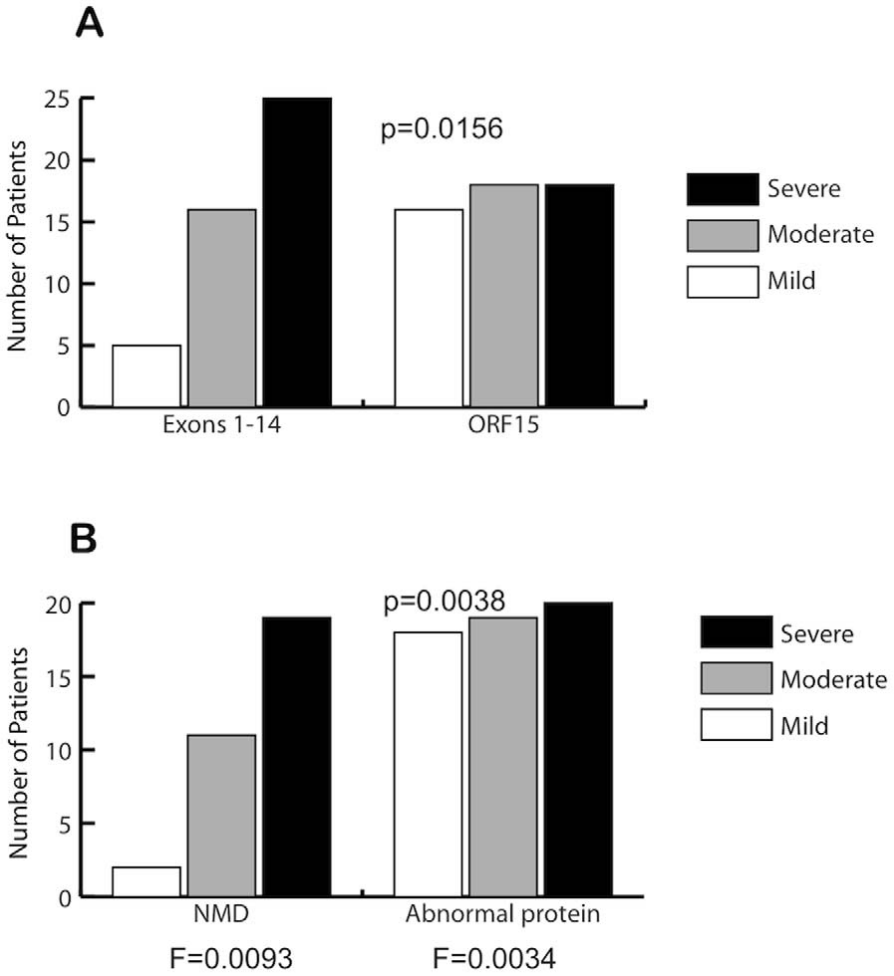
Allelic heterogeneity is associated with differences in clinical severity. The charts depict the distribution of clinical severity for (A) mutations in exons 1–14 vs. mutations in ORF15 and (B) mutations that are predicted to result in nonsense-mediated decay (NMD) vs. mutations that are predicted to result in a translated, abnormal protein product. Mild = grade 1; Moderate = grade 2; Severe = grade 3. P-values were calculated using a two-tailed Fisher Exact Test comparing grade 1 and grade 3 patients in each group (grade 2 patients were excluded). F is the average coefficient of inbreeding.

The 56 families carried 44 different mutations in *RPGR* (Table 2). Forty-six of the 98 affected males had mutations in exons 1–14, and 52 had mutations in ORF15. The distribution of clinical severity differed significantly between these two mutation groups (Figure 4A). Patients with mutations in exons 1–14 were more likely to be severely affected than patients with mutations in ORF15 (p = 0.016). The group with ORF15 mutations had a greater degree of phenotypic variability, with patients more evenly distributed among the 3 clinical categories.

**Table 2:** Mutations in 98 males from 56 families divided by location and mutation type.

Mutation Type	Mutation	Exon	# Patients	Grade 1(%)	Grade 2(%)	Grade 3(%)
Ex1–14 Missense	c.194G>A (p.G65D) [16]	3	2	0	1	1
	c.296C>T (p.T99I)	4	1	1	0	0
	c.617C>T (p.T206I)	6	1	0	0	1
	c.815G>A (p.G272D)	8	1	1	0	0
		**Total for Category:**		**2** (40)	**1** (20)	**2** (40)
Ex1–14 Splice Site	IVS2-1G>A [52]		3	1	2	0
	c.301_IVS4+3del		2	0	1	1
	IVS4+3A>G [53]		1	0	0	1
	IVS7-1G>A [12]		1	0	1	0
	IVS13-8 A>G [54]		1	0	0	1
	IVS13-1_1576delGAAACinsAA		1	0	0	1
		**Total for Category:**		**1** (12)	**4** (44)	**4** (44)
Ex1–14 Nonsense	c.730A>T (p.K244X) [55]	7	3	0	0	3
	c.838_842del (p.L280X) [12]	8	2	0	1	1
	c.851C>G (p.S284X)	8	1	0	1	0
	c.1126G>T (p.E376X)	10	2	0	0	2
		**Total for Category:**		**0** (0)	**2**(25)	**6** (75)
Ex1–14 Frameshift	c.101del (p.N34Mfs*34) [55]	2	10	0	2	8
	c.219del (p.A74Pfs*11)	3	1	0	1	0
	c.356del (p.L119Wfs*14) [56]	5	1	0	1	0
	c.372del (p.E125Kfs*8) [53]	5	1	0	0	1
	c.629_639del (p.E210Vfs*5) [57]	7	2	0	0	2
	c.1092dupA (p.A365Cfs*12)	10	1	0	0	1
	c.1243_1244del (p.R415Gfs*37)	10	2	0	2	0
	c.1319_1322del (p.C440Ffs*35)	11	2	0	2	0
	c.1377_1378del (p.L460Ifs*2) [58]	11	3	1	1	1
	c.1662_1665del (p.E555Gfs*14)	14	1	1	0	0
		**Total for Category:**		**2** (8)	**9** (38)	**13** (54)
		**TOTAL FOR EX1–14:**		**5**(11)	**16**(35)	**25**(54)
ORF15 Nonsense	ORF15+327A>T (ORF15K109X) [12]		1	0	0	1
	ORF15+393G>T (ORF15Glu129X) [57]		2	1	0	1
	ORF15+423G>T (ORF15Glu141X)		1	0	1	0
	ORF15+459G>T (ORF15G153X)		2	0	0	2
		**Total for Category:**		**1** (17)	**1** (17)	**4** (66)
ORF15 FS <50aa	ORF15+483_484del (ORF15E161Rfs*23) [7]		6	1	1	4
	ORF15+587del (ORF15A196Rfs*34) [12]		3	0	3	0
	ORF15+652_653del (ORF15E217Gfs*32) [7]		17	6	7	4
	ORF15+673_674del (ORF15E224Gfs*25) [7]		2	0	1	1
	ORF15+689_692del (ORF15G232fs*2) [7]		2	1	1	0
	ORF15+730_743del (ORF15E828Gfs*2)		1	1	0	0
		**Total for Category:**		**9** (29)	**13** (42)	**9** (29)
ORF15 FS>50aa	ORF15+185_186insAGAGG (ORF15A62Rfs*52) [57]		1	0	0	1
	ORF15+481_484del (ORF15R160Kfs*69) [12]		1	0	0	1
	ORF15+499_502del (ORF15K166fs*229) [57]		2	2	0	0
	ORF15+521_524del (ORF15G174Kfs*55)		1	0	1	0
	ORF15+720del (ORF15E240Rfs*264)		1	0	1	0
	ORF15+763_767del (ORF15E254Gfs*238) [12]		2	1	1	0
	ORF15 c.764_765del (ORF15E256Gfs*237) [12]		1	0	0	1
	ORF15+848_849del (ORF15E283Gfs*210) [59]		2	1	1	0
	ORF15+872dupA (ORF15G291Rfs*203) [7]		1	0	0	1
	ORF15+1038delG (ORF15E346R*158)		1	1	0	0
	ORF15+1114delA (ORF15E371Gfs*133) [16]		2	1	0	1
		**Total for Category:**		**6** (40)	**4** (27)	**5** (33)
		**TOTAL FOR ORF15:**		**16** (30)	**18**(35)	**18**(35)

FS<50aa = frameshift followed by fewer than 50 amino acids before termination, and FS>50aa = frameshift followed by more than 50 amino acids before termination. Mutations without a reference were detected in the Daiger laboratory.

Patients were regrouped according to whether the mutation was predicted to result in a translated protein product. Premature stop codons in exons 1–14, or frameshift mutations in exons 1–14 leading to premature stop codons, are predicted to result in nonsense-mediated decay of the transcript and are therefore predicted null alleles. ORF15, by contrast, is the terminal exon of the ORF15 variant transcript, and therefore nonsense mutations and frameshifts in ORF15 may result in a stable transcript which is translated into a variant protein product. Missense mutations are also predicted to result in translated variant proteins. Splice site mutations are unpredictable in their effects on transcription and translation since they may eliminate the transcript, may eliminate an exon, may favor a cryptic splice site, may shift the fraction of alternate transcripts, and/or may occasionally produce a correctly-spliced transcript. For this reason, splice-site variants were excluded from the analysis.

Figure 4B shows a striking difference in clinical severity between these two groups, similar to the difference observed based on mutation location. Predicted null alleles were associated with more severe disease, while alleles predicted to result in variant protein demonstrated more phenotypic variability (p = 0.0038). The average coefficient of inbreeding (F) was 0.0093 for the group with predicted null alleles and 0.0034 for the group with predicted variant protein (Fig. 4B). The increased relatedness in the former group indicates that shared genetic factors other than the disease-causing mutation may account for the increased clinical severity. If only individuals separated by at least 4 meioses are included (sharing ≤6.25% of their DNA), then the difference between mutations in exons 1–14 and ORF15 is suggestive but not significant (p = 0.083), while the difference between predicted null alleles and predicted translated protein alleles remains significant (p = 0.049). In addition, generalized estimating equation (GEE) analysis was used to further assess the difference in disease severity between patients with predicted null alleles and patients with predicted translated protein, and confirmed that the presence of a predicted null *RPGR* allele is associated with more severe disease (p = 0.040).

### RPGRIP1 and RPGRIP1L protein haplotypes

When considering polymorphic variation in a protein, the protein haplotypes may be more biologically relevant than individual amino acid substitutions. We aimed to determine the common protein haplotypes in two of our candidate modifiers in order to fulfill two goals, 1) to determine the degree of polymorphic variation in the candidate modifier proteins, and 2) to assist in determining phase when coding SNPs were genotyped in patients.

In order to find common protein haplotypes of RPGRIP1 and RPGRIP1L, we used DNA from murine-human hybrid cell lines (a gift from Dr. Jim Hixson) that were monosomic for either human chromosome 14 or 16, including the *RPGRIP1* and *RPGRIP1L* genes, respectively [43]. Coding SNPs from dbSNP and the literature [22], [26] were sequenced in at least 100 monosomic cell lines, including 32–38 lines derived from each of three ethnicities: African-American, European-American, and Mexican-American (Tables 3 and 4). Far more variation was found in RPGRIP1, with 14 different polymorphic protein haplotypes and a frequency of 41% for the most common haplotype, compared to only 5 different protein haplotypes for RPGRIP1L and a frequency of 85% for the most common. Note that for each protein, the most common haplotype overall is also the most common haplotype within each ethnicity. However, there were also striking differences among ethnicities. In particular, RPGRIP1 haplotype #9 is the second most common haplotype in European-Americans at 25%, but had frequencies of only 2.9% and 0% in Mexican-Americans and African-Americans, respectively.

**Table 3:** Common protein haplotypes of RPGRIP1.

Haplotype	P96Q (ex 3)	K192E (ex 4)	A547S (ex 13)	R598Q (ex 14)	A960P (ex 17)	E1033Q (ex 18)	D1114G (ex 21)	D1150T (ex 21)	G1240E (ex 23)	Overall freq	AA freq	EA freq	MA freq
1	Pro	Lys	Ala	Arg	Ala	Glu	Asp	Asp	Gly	**0.410**	0.485	0.375	0.371
2	**Gln**	**Glu**	Ala	Arg	Ala	Glu	Asp	Asp	Gly	**0.150**	0.091	0.031	0.314
3	**Gln**	**Glu**	Ala	**Gln**	Ala	Glu	Asp	Asp	**Glu**	**0.02**	0.061	0	0
4	Pro	**Glu**	Ala	**Gln**	Ala	Glu	Asp	Asp	**Glu**	**0.02**	0.061	0	0
5	Pro	**Glu**	Ala	Arg	Ala	Glu	Asp	Asp	Gly	**0.090**	0.091	0	0.171
6	Pro	**Glu**	Ala	Arg	Ala	Glu	Asp	**Tyr**	**Glu**	**0.01**	0.03	0	0
7	Pro	**Glu**	**Ser**	Arg	Ala	Glu	Asp	Asp	**Glu**	**0.01**	0.03	0	0
8	Pro	**Glu**	**Ser**	Arg	Ala	Glu	Asp	Asp	Gly	**0.070**	0.061	0.125	0.029
9	Pro	**Glu**	**Ser**	Arg	Ala	**Gln**	Asp	Asp	Gly	**0.090**	0	0.250	0.029
10	Pro	**Glu**	Ala	Arg	Ala	**Gln**	Asp	Asp	Gly	**0.080**	0	0.156	0.086
11	Pro	Lys	Ala	Arg	Ala	**Gln**	Asp	Asp	Gly	**0.02**	0	0.063	0
12	Pro	Lys	**Ser**	Arg	Ala	Glu	Asp	Asp	Gly	**0.01**	0.03	0	0
13	Pro	Lys	Ala	Arg	**Pro**	Glu	Asp	Asp	Gly	**0.01**	0.03	0	0
14	Pro	Lys	Ala	Arg	Ala	Glu	**Gly**	Asp	Gly	**0.01**	0.03	0	0

Protein haplotypes from over 100 cell lines monosomic for chromosome 14, and their frequencies. Minor alleles are in bold. Ex = exon, freq = frequency, AA = African American, EA = European American, MA = Mexican American.

**Table 4:** Common protein haplotypes of RPGRIP1L.

Haplotype	A229T (ex 6)	R744Q (ex 16)	G1025S (ex 21)	D1264N (ex 26)	Overall frequency	AA freq	EA freq	MA freq
**1**	Ala	Arg	Gly	Asp	**0.851**	0.842	0.897	0.811
**2**	**Thr**	Arg	**Ser**	Asp	**0.053**	0.053	0	0.108
**3**	Ala	Arg	**Ser**	Asp	**0.035**	0.079	0	0.027
**4**	Ala	Arg	**Ser**	**Asn**	**0.026**	0	0.026	0.054
**5**	Ala	**Gln**	Gly	Asp	**0.035**	0.026	0.077	0

Protein haplotypes from over 100 cell lines monosomic for chromosome 16, and their frequencies. Minor alleles are in bold. Ex = exon, freq = frequency, AA = African American, EA = European American, MA = Mexican American.

### Modifier SNPs

Common coding SNPs (MAF≥2%) in *RPGRIP1*, *RPGRIP1L*, *CEP290*, and *IQCB1* (a.k.a. *NPHP5*) were sequenced in all 98 affected males as well as 99 available family members. Family-based association testing was performed using PLINK [46] comparing grade 1 and grade 3 RP cases. Analysis was performed on 1) the entire cohort, 2) the subgroup with predicted null RPGR alleles, 3) the subgroup with predicted variant RPGR protein, 4) the subgroup with mutations in exons 1–14, and 5) the subgroup with mutations in ORF15 (Table 5). The PLINK output data is presented in table format in Tables S1, S2, S3, S4, S5.

**Table 5:** Two candidate modifier loci are associated with disease severity in the cohort.

Gene:	*RPGRIP1*				*RPGRIP1L*				*CEP290*		*IQCB1*	
cSNP:	P96Q	K192E	A547S	E1033Q	A229T	R744Q	G1025S	D1264N	K838E	L906W	I393N	C434Y
P value all	0.6224	0.3666	0.6835	0.2943	0.5637	**0.0493**	0.5227	0.9196	0.6984	NA	**0.0438**	0.9774
P value protein	0.0768	0.3721	0.2230	0.3018	0.5637	0.2444	0.2383	0.2677	0.3483	NA	1.0000	0.4239
P value null	0.0782	0.0834	0.1195	0.0770	NA	0.4551	0.7288	0.2572	0.1883	NA	**0.0298**	0.4870
P value ex 1–14	0.0707	**0.0358**	0.2658	**0.0455**	0.3173	0.3046	0.6687	0.5909	0.3046	NA	**0.0176**	0.2353
P value ORF15	0.0768	0.1706	0.0737	0.2528	1.0000	0.2444	0.6749	0.5318	0.3483	NA	1.0000	0.2296

Common coding SNPs in candidate modifier genes were tested for association with disease severity in the total cohort (“all” in left column) and in the subgroups with predicted translated RPGR protein (“protein” in left column), with predicted null alleles (“null” in left column), with exon 1–14 mutations (“ex 1–14” in left column), and with ORF15 mutations (“ORF15” in left column). P-values<0.05 are shown in bold.

In the entire cohort, two coding SNPs showed significant association with disease severity: rs1141528 in *IQCB1*, which results in substitution of asparagine for isoleucine at codon 393 (I393N), and rs2302677 in *RPGRIP1L*, which results in substitution of glutamine for arginine at codon 744 (R744Q). In *IQCB1*, the minor allele (asparagine) at codon 393 was associated with more severe disease (p = 0.044), while in *RPGRIP1L*, the common allele (arginine) at codon 744 was associated with more severe disease (p = 0.049). In the group with RPGR mutations predicted to result in a translated protein product, no SNPs showed significant association with disease severity. However, in the group with predicted null *RPGR* alleles, the association between the asparagine at codon 393 (I393N) in *IQCB1* increased in significance (p = 0.030). Association testing was also performed on groups divided by mutation location: exons 1–14 vs. ORF15. In the ORF15 group, which has extensive overlap with the group predicted to result in a translated protein product, no SNPs showed significant association with disease severity. In the exons 1–14 group, the minor allele at I393N in *IQCB1* again showed association with disease severity, increased in significance compared to the total cohort (p = 0.018). Two SNPs in *RPGRIP1* which were not associated with disease severity in the total cohort showed significant association in the exons 1–14 group: the minor allele at K192E (p = 0.036) and the minor allele at E1033Q (0.046).

## Discussion

This study aimed to categorize the phenotypic variation in affected males with XlRP from 52 families carrying mutations in *RPGR*, and to determine the contribution of allelic heterogeneity and genetic modifiers to this variation. We report considerable variation in clinical severity both between and within families. A significant genotype-phenotype correlation was observed: individuals with mutations in exons 1–14 are more likely to have severe disease than individuals with mutations in ORF15, which is consistent with previous observations [12]. Furthermore, individuals with predicted null alleles are also more likely to have severe disease in comparison to the group predicted to have variant RPGR proteins, which demonstrates more phenotypic diversity.

A number of studies support the predication that a premature termination mutation in other than the final exon of a gene results in nonsense-mediated decay [47], [48]. For example, patients with the *RPGR* Q236X nonsense variant have non-detectable RPGR protein by western blot in lymphoblasts, while control lymphoblasts demonstrate both RPGR_1–19_ and RPGR_ORF15_ protein isoforms [49]. However, categorization of mutant alleles into predicted nulls vs. predicted translated proteins is based on our current understanding of nonsense-mediated decay and may not reflect the biologic presence or absence of protein translation in every case, nor specifically within the retina. Nonetheless, these findings suggest that expression of variant RPGR protein is conducive to variable expressivity of disease while the absence of protein more uniformly causes severe disease.

Candidate modifier loci were chosen on the basis of previously demonstrated protein-protein interaction with RPGR, known retinal disease-causing mutations, and known common protein variation. RPGRIP1 and RPGRIP1L have been shown to interact with RPGR in yeast 2-hybrid and co-immunoprecipitation experiments [23], [26], and both proteins localize to the photoreceptor connecting cilium [24], [26]. Mutations in *RPGRIP1* cause Leber congenital amaurosis (LCA6 [MIM 605446]) and cone-rod dystrophy (CORD13 [MIM 608194]) [22], [25], and mutations in *RPGRIP1L* cause Joubert syndrome (JBTS7 [MIM 611560]) and Meckel syndrome (MKS5 [MIM 611561]) [27], [28], two syndromic ciliopathies with associated retinal dystrophy. CEP290 and IQCB1 have shown interaction with RPGR by co-immunoprecipitation and both localize to the photoreceptor connecting cilium [29], [37], while IQCB1 also localizes to the primary cilia in renal epithelial cells [37]. Mutations in *CEP290* cause Joubert syndrome (JBTS5 [MIM 610188]), Meckel syndrome (MKS4 [MIM 611134]), Senior Loken syndrome (SLSN6 [MIM 610189]), Leber congenital amaurosis (LCA10 [MIM 611755]) [30], [31], [32], [33], [34], [35], and Bardet Biedel syndrome (BBS14 [MIM 209900]) [36], while mutations in *IQCB1* are the most frequent cause of Senior Loken syndrome (SLSN5 [MIM 609254]) [37], a syndromic disease with both nephronophthisis and retinitis pigmentosa.

Two coding SNPs in candidate modifier genes showed marginally significant association with disease severity in our cohort: the asparagine (N) allele of I393N in *IQCB1* and the arginine (R) allele of R744Q in *RPGRIP1L*. We hypothesized that patients with no RPGR protein may be susceptible to different modifying effects than patients with variant RPGR protein, and therefore we separated the cohort into patients predicted to have RPGR protein and those predicted to have no RPGR protein and recalculated the SNP associations. While no SNPs showed significant association in the subgroup with predicted RPGR protein, the N allele of I393N in *IQCB1* was associated with severity in the group predicted to have no RPGR protein, and this association was more significant in the subgroup than in the total cohort. Furthermore, this same SNP also showed significant association with disease severity in the subgroup of patients with mutations in exons −14.

Polyphen-2 (http://genetics.bwh.harvard.edu/pph2/) [50] predicts the N allele in I393N to be benign while the R allele in R744Q is predicted to be probably damaging, with a difference in position-specific independent count (PSIC) score of 0.94. Amino acid residue 393 in IQCB1 lies within one of two calmodulin-binding domains, and IQCB1 has demonstrated interaction with calmodulin-2 by yeast 2-hybrid experiments and co-immunoprecipitation from retinal extracts [37]. Calmodulin has been shown to modulate the rod cGMP-gated cation channel mediating rod visual transduction [51]. Amino acid residue 744 in RPGRIP1L lies between the two C2 domains (PKC conserved region 2 motif) [28].

The more C-terminal C2 domain of RPGRIP1L is responsible for binding to nephrocystin-4, and mutations in *NPHP4* which cause Senior Loken Syndrome disrupt this interaction with RPGRIP1L [27]. Studies have shown that the carboxyl-terminus of RPGRIP1L is responsible for binding to RPGR, although the boundaries of the RPGR-binding domain have not been defined [26]. Given the location of the RPGR-binding domain in RPGRIP1, which is 29% identical to RPGRIP1L at the amino acid level and shares the same general domain structure [27], it is reasonable to postulate that amino acid 744 in RPGRIP1L lies *outside* the RPGR-binding domain.

Both modifier loci identified in this study showed marginally significant association with severity. Future studies should include genotyping these SNPs in additional males with *RPGR* mutations to either confirm or refute these findings, in addition to functional experiments to investigate a biologic mechanism for the modifying effect. As with all association studies, it is possible that a coding SNP showing association is not the true modifier locus, but is in linkage disequilibrium with the causal SNP. This is especially relevant if the true modifier is a common non-coding SNP, since we only tested coding SNPs with MAF≥2%. On the other hand, a single rare variant would not be sufficient to explain the high frequency of severe and mild cases, and a series of rare variants in aggregate should not be in disequilibrium with a single common variant.

A common variant in the RPGRIP1L protein, A229T, was recently reported to be associated with the presence of retinopathy in a cohort of syndromic ciliopathy patients [26]. The current study does not demonstrate significant association between the A229T variant and clinical severity in males with *RPGR* mutations. However, our analysis of common RPGRIP1L protein haplotypes in monosomic cell lines revealed that the A229T variant occurred on the background of the G1025S variant and that these 2 loci are in linkage disequilibrium. While G1025S is frequently present without A229T, the converse was not observed.

Analysis of protein haplotypes is important in our study, because the protein haplotype is the true “allele” that interacts with RPGR and therefore has the most biologic relevance when considering protein-protein interactions. However, association testing of protein haplotypes did not reveal any significant associations in our cohort. This may be in part due to sample size: while SNP analysis is bi-allelic, haplotype analysis is multi-allelic and therefore the analysis includes fewer patients for each haplotype “allele.”

The discovery of modifier genes provides new avenues of investigation regarding both disease biology and potential therapeutics. In addition, modifier genes have potential prognostic utility. While individual genetic modifier loci may have a small impact on disease severity on their own, the goal is to continue defining the total genetic contribution to disease in order to build a more complete prognostic toolset. This includes both the underlying Mendelian mutations, as well as genetic modifiers influencing phenotypic expression. This study demonstrates a contribution from both of these types of variables to phenotypic heterogeneity.

## Supporting Information

Table S1 **Output data from PLINK Dfam analysis of SNP association with disease severity in all grade 1 and 3 patients.** CHR = chromosome number, SNP = SNP identifier, A1 = minor allele, A2 = major allele, OBS = number of observed minor alleles, EXP = number of expected minor alleles, CHISQ = Chi-squared test statistic, P = asymptotic p-value.
(DOC)

Table S2 **Output data from PLINK Dfam analysis of SNP association with disease severity in grade 1 and 3 patients predicted to make a translated RPGR protein.** CHR = chromosome number, SNP = SNP identifier, A1 = minor allele, A2 = major allele, OBS = number of observed minor alleles, EXP = number of expected minor alleles, CHISQ = Chi-squared test statistic, P = asymptotic p-value.
(DOCX)

Table S3 **Output data from PLINK Dfam analysis of SNP association with disease severity in grade 1 and 3 patients predicted to have a null RPGR allele.** CHR = chromosome number, SNP = SNP identifier, A1 = minor allele, A2 = major allele, OBS = number of observed minor alleles, EXP = number of expected minor alleles, CHISQ = Chi-squared test statistic, P = asymptotic p-value.
(DOCX)

Table S4 **Output data from PLINK Dfam analysis of SNP association with disease severity in grade 1 and 3 patients with mutations in RPGR ORF15.** CHR = chromosome number, SNP = SNP identifier, A1 = minor allele, A2 = major allele, OBS = number of observed minor alleles, EXP = number of expected minor alleles, CHISQ = Chi-squared test statistic, P = asymptotic p-value.
(DOC)

Table S5 **Output data from PLINK Dfam analysis of SNP association with disease severity in grade 1 and 3 patients with mutations in RPGR exons 1–14.** CHR = chromosome number, SNP = SNP identifier, A1 = minor allele, A2 = major allele, OBS = number of observed minor alleles, EXP = number of expected minor alleles, CHISQ = Chi-squared test statistic, P = asymptotic p-value.
(DOC)
