# Supplementary material for: Allelic Heterogeneity and Genetic Modifier Loci Contribute to Clinical Variation in Males with X-Linked Retinitis Pigmentosa Due to RPGR Mutations
Source: PLoS One. 2011 Aug 12;6(8):e23021. doi: 10.1371/journal.pone.0023021 (PMC3155520; doi:10.1371/journal.pone.0023021)
Supplement: Table S1 — Output data from PLINK Dfam analysis of SNP association with disease severity in all grade 1 and 3 patients. CHR = chromosome number, SNP = SNP identifier, A1 = minor allele, A2 = major allele, OBS = number of observed minor alleles, EXP = number of expected minor alleles, CHISQ = Chi-squared test statistic, P = asymptotic p-value. (DOC) [file pone.0023021.s001.doc]

| CHR | SNP | A1 | A2 | OBS | EXP | CHISQ | P |
| --- | --- | --- | --- | --- | --- | --- | --- |
| 3 | rs17849995 C434Y | A | G | 17 | 16.95 | 0.000805 | 0.9774 |
| 3 | rs1141528 I393N | A | T | 7 | 4.25 | 4.065 | 0.04379 |
| 12 | rs7970228 | C | A | 0 | 0 | NA | NA |
| 12 | rs11104738 | G | A | 4 | 3.6 | 0.1501 | 0.6984 |
| 14 | P96Q | A | C | 5 | 5.55 | 0.2425 | 0.6224 |
| 14 | K192E | G | A | 25 | 22.8 | 0.8152 | 0.3666 |
| 14 | A547S | T | G | 9 | 9.9 | 0.1662 | 0.6835 |
| 14 | rs3748361 E1033Q | C | G | 18 | 15.7 | 1.1 | 0.2943 |
| 16 | rs3213758 D1264N | A | G | 2 | 2.1 | 0.01018 | 0.9196 |
| 16 | rs2111119 G1025S | A | G | 5 | 4.1 | 0.4086 | 0.5227 |
| 16 | rs2302677 R744Q | A | G | 0 | 1.65 | 3.865 | 0.04931 |
| 16 | rs61747071 A229T | A | G | 2 | 1.5 | 0.3333 | 0.5637 |
