# Supplementary material for: Allelic Heterogeneity and Genetic Modifier Loci Contribute to Clinical Variation in Males with X-Linked Retinitis Pigmentosa Due to RPGR Mutations
Source: PLoS One. 2011 Aug 12;6(8):e23021. doi: 10.1371/journal.pone.0023021 (PMC3155520; doi:10.1371/journal.pone.0023021)
Supplement: Table S2 — Output data from PLINK Dfam analysis of SNP association with disease severity in grade 1 and 3 patients predicted to make a translated RPGR protein. CHR = chromosome number, SNP = SNP identifier, A1 = minor allele, A2 = major allele, OBS = number of observed minor alleles, EXP = number of expected minor alleles, CHISQ = Chi-squared test statistic, P = asymptotic p-value. (DOCX) [file pone.0023021.s002.docx]

| CHR | SNP | A1 | A2 | OBS | EXP | CHISQ | P |
| --- | --- | --- | --- | --- | --- | --- | --- |
| 3 | rs17849995 C434Y | A | G | 5 | 6.2 | 0.6393 | 0.4239 |
| 3 | rs1141528 I393N | A | T | 2 | 2 | 0 | 1 |
| 12 | rs7970228 L906W | C | A | 0 | 0 | NA | NA |
| 12 | rs11104738 K838E | G | A | 0 | 0.6 | 0.8797 | 0.3483 |
| 14 | P96Q | A | C | 2 | 0.8 | 3.13 | 0.07684 |
| 14 | K192E | G | A | 7 | 8.8 | 0.7965 | 0.3721 |
| 14 | A547S | T | G | 3 | 5.4 | 1.485 | 0.223 |
| 14 | rs3748361 E1033Q | C | G | 6 | 7.7 | 1.066 | 0.3018 |
| 16 | rs3213758 D1264N | A | G | 2 | 1.1 | 1.229 | 0.2677 |
| 16 | rs2111119 G1025S | A | G | 4 | 2.6 | 1.391 | 0.2383 |
| 16 | rs2302677 R744Q | A | G | 0 | 0.9 | 1.355 | 0.2444 |
| 16 | rs61747071 A229T | A | G | 2 | 1.5 | 0.3333 | 0.5637 |
