# Supplementary material for: Allelic Heterogeneity and Genetic Modifier Loci Contribute to Clinical Variation in Males with X-Linked Retinitis Pigmentosa Due to RPGR Mutations
Source: PLoS One. 2011 Aug 12;6(8):e23021. doi: 10.1371/journal.pone.0023021 (PMC3155520; doi:10.1371/journal.pone.0023021)
Supplement: Table S3 — Output data from PLINK Dfam analysis of SNP association with disease severity in grade 1 and 3 patients predicted to have a null RPGR allele. CHR = chromosome number, SNP = SNP identifier, A1 = minor allele, A2 = major allele, OBS = number of observed minor alleles, EXP = number of expected minor alleles, CHISQ = Chi-squared test statistic, P = asymptotic p-value. (DOCX) [file pone.0023021.s003.docx]

| CHR | SNP | A1 | A2 | OBS | EXP | CHISQ | P |
| --- | --- | --- | --- | --- | --- | --- | --- |
| 3 | rs17849995 C434Y | A | G | 9 | 9.85 | 0.4831 | 0.487 |
| 3 | rs1141528 I393N | A | T | 4 | 1.75 | 4.722 | 0.02978 |
| 12 | rs7970228 L906W | C | A | 0 | 0 | NA | NA |
| 12 | rs11104738 K838E | G | A | 4 | 2.8 | 1.731 | 0.1883 |
| 14 | P96Q | A | C | 3 | 4.65 | 3.103 | 0.07817 |
| 14 | K192E | G | A | 12 | 9.4 | 2.997 | 0.0834 |
| 14 | A547S | T | G | 4 | 2.2 | 2.423 | 0.1195 |
| 14 | rs3748361 E1033Q | C | G | 7 | 4.1 | 3.126 | 0.07703 |
| 16 | rs3213758 D1264N | A | G | 0 | 0.8 | 1.284 | 0.2572 |
| 16 | rs2111119 G1025S | A | G | 1 | 1.3 | 0.1202 | 0.7288 |
| 16 | rs2302677 R744Q | A | G | 0 | 0.45 | 0.558 | 0.4551 |
| 16 | rs61747071 A229T | A | G | 0 | 0 | NA | NA |
