# Supplementary material for: Allelic Heterogeneity and Genetic Modifier Loci Contribute to Clinical Variation in Males with X-Linked Retinitis Pigmentosa Due to RPGR Mutations
Source: PLoS One. 2011 Aug 12;6(8):e23021. doi: 10.1371/journal.pone.0023021 (PMC3155520; doi:10.1371/journal.pone.0023021)
Supplement: Table S5 — Output data from PLINK Dfam analysis of SNP association with disease severity in grade 1 and 3 patients with mutations in RPGR exons 1–14. CHR = chromosome number, SNP = SNP identifier, A1 = minor allele, A2 = major allele, OBS = number of observed minor alleles, EXP = number of expected minor alleles, CHISQ = Chi-squared test statistic, P = asymptotic p-value. (DOC) [file pone.0023021.s005.doc]

| **CHR** | **SNP** | **A1** | **A2** | **OBS** | **EXP** | **CHISQ** | **P** |
| --- | --- | --- | --- | --- | --- | --- | --- |
| 3 | rs17849995 C434Y | A | G | 14 | 12.25 | 1.408 | 0.2353 |
| 3 | rs1141528 I393N | A | T | 5 | 2.25 | 5.638 | 0.01758 |
| 12 | rs7970228 L906W | C | A | 0 | 0 | NA | NA |
| 12 | rs11104738 K838E | G | A | 4 | 3 | 1.054 | 0.3046 |
| 14 | P96Q | A | C | 3 | 4.75 | 3.267 | 0.0707 |
| 14 | K192E | G | A | 20 | 15.5 | 4.406 | 0.03582 |
| 14 | A547S | T | G | 8 | 6 | 1.238 | 0.2658 |
| 14 | rs3748361 E1033Q | C | G | 14 | 10 | 4 | 0.0455 |
| 16 | rs3213758 D1264N | A | G | 1 | 1.5 | 0.2889 | 0.5909 |
| 16 | rs2111119 G1025S | A | G | 3 | 2.5 | 0.1831 | 0.6687 |
| 16 | rs2302677 R744Q | A | G | 0 | 0.75 | 1.054 | 0.3046 |
| 16 | rs61747071 A229T | A | G | 1 | 0.5 | 1 | 0.3173 |
